# What Interventions Work to Reduce Cost Barriers to Primary Healthcare in High-Income Countries? A Systematic Review

**DOI:** 10.3390/ijerph21081029

**Published:** 2024-08-05

**Authors:** Bailey Yee, Nisa Mohan, Fiona McKenzie, Mona Jeffreys

**Affiliations:** Te Hikuwai Rangahau Hauora, Health Services Research Centre, Te Herenga Waka–Victoria University of Wellington, Wellington 6011, New Zealand; bailey.yee@vuw.ac.nz (B.Y.); nisa.mohan@vuw.ac.nz (N.M.); fiona.mckenzie@vuw.ac.nz (F.M.)

**Keywords:** cost barriers, health expenditures, health services accessibility, primary healthcare

## Abstract

High-income countries like Aotearoa New Zealand are grappling with inequitable access to healthcare services. Out-of-pocket payments can lead to the reduced use of appropriate healthcare services, poorer health outcomes, and catastrophic health expenses. To advance our knowledge, this systematic review asks, “What interventions aim to reduce cost barriers for health users when accessing primary healthcare in high-income countries?” The search strategy comprised three bibliographic databases (Dimensions, Embase, and Medline Web of Science). Two authors selected studies for inclusion; discrepancies were resolved by a third reviewer. All articles published in English from 2000 to May 2022 and that reported on outcomes of interventions that aimed to reduce cost barriers for health users to access primary healthcare in high-income countries were eligible for inclusion. Two blinded authors independently assessed article quality using the Critical Appraisal Skills Program. Relevant data were extracted and analyzed in a narrative synthesis. Forty-three publications involving 18,861,890 participants and 6831 practices (or physicians) met the inclusion criteria. Interventions reported in the literature included removing out-of-pocket costs, implementing nonprofit organizations and community programs, additional workforce, and alternative payment methods. Interventions that involved eliminating or reducing out-of-pocket costs substantially increased healthcare utilization. Where reported, initiatives generally found financial savings at the system level. Health system initiatives generally, but not consistently, were associated with improved access to healthcare services.

## 1. Introduction

Primary healthcare constitutes an integral part of the health system and is widely regarded as the most inclusive and cost-effective strategy to achieve universal health coverage [1]. As it is often the first point of contact with the health system and a gatekeeper to secondary services, access to primary healthcare is a critical factor in the performance of the whole health system [2]. Access to primary healthcare refers to the attributes of the service provided, the characteristics of health professionals, and the resources used to enable a population to seek and obtain care [2]. It is suggested by the World Health Organization and other health agencies that a primary healthcare system that is robust and accessible is paramount to reducing healthcare inequities, improving system efficiency, and achieving better patient experience and quality of treatment [1,3,4].

Many high-income countries, including the United Kingdom, Italy, Australia, Canada, and Aotearoa New Zealand, employ a capitation formula to fund primary healthcare and/or secondary services. This formula accounts for the size and characteristics of a population, such as age and sex, to estimate the funding needed to match the community’s predicted healthcare needs [5]. However, these formulae are primarily modelled on historical healthcare utilization and insufficiently account for the issues experienced through and between heterogeneous population groups, such as deprivation and ethnicity, which often reinforce health disparities and inequities [2,6]. In health systems like those in Australia and Aotearoa New Zealand, consultation costs that exceed the capitation coverage are paid out of pocket by the health user or guardian. International evidence reports that relying on out-of-pocket healthcare payments is inequitable and inefficient [7]. The arguments for copayments are that they promote cost-conscious service utilization and may encourage healthier lifestyle behaviours [8]. Nonetheless, when healthcare is appropriate, copayments can delay treatment, resulting in health status deterioration and increased financial burden for the overall system, for example, higher rates of avoidable hospital admission [7,9]. A large-scale, multiyear experience found that cost sharing (i.e., costs are shared by insurance and paid out of pocket by a user) reduced “inappropriate or unnecessary” medical care but also, subsequently, “appropriate or necessary” healthcare services [9]. Furthermore, out-of-pocket costs disproportionately affect those of lower socioeconomic status and exacerbate poverty [10]. It is argued that out-of-pocket costs for primary healthcare services are a cause of or, at the very least, an aggravator of poverty [11].

In October 2015, the United Nations adopted the Sustainable Development Goals (SDGs) to stimulate action over the next fifteen years in areas of critical significance for humanity and the planet [12]. Of importance, SDG3 (Target 3.8) set the objective for Member States to achieve universal health coverage, including protection against financial risk and the accessibility to medicines and vaccines for all [13]. Despite the commitment from Member States, primary healthcare systems worldwide still need to be developed, as they fail to provide high-quality, inclusive, affordable services for all populations.

It is more necessary than ever to address the inaccessibility and unaffordability of primary healthcare services in high-income countries. The consequences of failing to invest in primary care were demonstrated in the COVID-19 pandemic, which, in turn, brought a renewed focus on a primary healthcare system that is robust and affordable. Various strategies, with differing levels of success, have been implemented internationally to reduce financial barriers to the health user when accessing primary healthcare. This has led to the implementation of a diverse range of programs that intend to improve access to healthcare services but more often create confusion for the health user and clutter the healthcare space. Thus, we conducted a systematic literature review to synthesize evidence of interventions addressing cost barriers for health users when accessing primary healthcare in high-income countries. From this, we aimed to comprehensively review successful strategies that may benefit other high-income countries.

## 2. Materials and Methods

This systematic review was conducted and reported following the Preferred Reporting Items for Systematic Reviews and Meta-analysis (PRISMA) Guidelines. Given the heterogeneity of the study designs included in this review, no meta-analysis was undertaken. Instead, we conducted a narrative synthesis and applied a quality appraisal using the Critical Appraisal Skills Program (CASP) checklists. No protocol was registered for this review.

### 2.1. Search Strategy and Retrieval of Studies

Searches of databases using a combination of key terms were trialed in April 2022. The purpose of this trial was to provide evidence of which terms offered the most comprehensive retrieval of the literature and informed the selection of databases used within the search strategy. Three electronic bibliographic databases were selected as a result of the trialing process: Medline Web of Science, EMBASE, and Dimensions. A preliminary search found that few publications included relevant terms within the title or abstract. Therefore, the search for keywords included the full text of the article to allow for a substantial collation of evidence to undergo eligibility screening.

Limits applied to the search were tailored to each database. For example, Dimensions allowed countries to be selected to provide publications from high-income countries. However, not all high-income countries, as defined by World Bank Data [14], were available to choose from. Therefore, we accounted for this by including “NOT low/medium income countr*”. This strategy was also applied to the Web of Science and EMBASE. Furthermore, all databases allowed us to limit our search to English-only publications and exclude specific research studies, such as literature reviews, study protocols, and presentations.

The search of the literature using the three electronic databases and predefined search terms was conducted in April 2022. Research keywords are outlined in Table 1 and Table 2. Search terms from the combined databases were imported into Zotero, a bibliographic management program, and duplicate citations were removed using Zotero’s duplicate identification tool [15]. Duplicates that were missed by Zotero were highlighted by Rayaan’s duplication identification tool later in the eligibility screening. An additional search of reference lists of single studies and literature reviews that were deemed relevant but that did not fit the eligibility criteria was undertaken.

### 2.2. Inclusion and Exclusion Criteria

The eligibility criteria of this systematic review included the following:

1. The article reported on the outcomes of interventions;
2. Interventions aimed to reduce cost barriers in primary healthcare settings;
3. The study included a defined population residing in a high-income country, as defined by the World Bank;
4. The article was published in English between January 2000 and April 2022.

Peer-reviewed articles for all study designs, including observational and experimental, were eligible for inclusion. Exclusion criteria included articles published before January 2000, non-English-language studies, and those on low- to medium-income countries. The exclusion of low- to medium-income countries was conducted by omitting any articles that mentioned “low-income country(/ies)” or “medium-income country(/ies)” in their title or abstract. No specific countries were individually named to be excluded.

### 2.3. Study Selection

The remaining publications were exported from Zotero into Rayaan, an online tool designed to facilitate the screening and selection of studies [16]. Two reviewers (BY and NM) independently undertook an initial screening by title and abstract. A third reviewer (FMcK or MJ) adjudicated conflicting records to ensure uniformity. If the title and/or abstract of a potentially relevant citation did not provide sufficient information to determine inclusion, the report proceeded to the full-text review, in which two reviewers (BY and NM, FMcK, or MJ) independently assessed the article’s manuscript for eligibility.

### 2.4. Data Extraction

Data from articles were extracted by one reviewer (BY) and checked by another (NM, FMcK, or MJ), with any discrepancies resolved through discussion. The following information from quantitative and qualitative studies was extracted using a template in Microsoft Excel: (1) publication details (i.e., author, year, and country); (2) methods (i.e., population, study design, and data); (3) intervention and comparator; (4) results and outcomes.

### 2.5. Quality of Evidence

Two reviewers critically appraised all included publications using the CASP checklists (see Supplementary Tables S1–S4). In most studies, reviewers agreed on the articles’ appraisal and quality assessment determinations. Where discrepancies appeared, these were resolved by the resolution of one experienced reviewer (MJ). As recommended by the CASP checklist, the items listed were not scored using a point system. Instead, items were recorded using “yes”, “no” or “not applicable” [N/A] responses. The accumulation of these responses determined the overall appraisal of the publication as “satisfactory” or included with “caution” (see Table 3, Table 4, Table 5, Table 6, Table 7 and Table 8).

### 2.6. Synthesis Process

Given the heterogeneity of study designs, we combined the results in a narrative synthesis. Study findings were tabulated into categorized intervention types (i.e., total removal of out-of-pocket payments), then grouped into outcome categories considered relevant, for example:

- Healthcare utilization: rates of physician visits.
- Medication adherence: improved, maintained, or discontinued pharmaceutical use.
- Cost savings: changes in out-of-pocket costs and expenditures per patient per practice.
- Accessibility: the likelihood of being accepted as a new patient by a healthcare provider and reported barriers experienced by a patient.

Interventions with more than one component (i.e., the Affordable Care Act [ACA] expansion with eligible/non-eligible dual beneficiaries) were categorized according to the primary component emphasized by the authors.

A subgroup analysis was conducted to synthesize evidence of interventions from high-income countries that employed a capitation-style funding arrangement for primary healthcare service providers. This allowed for an examination of the cost-reducing methods most complementary to the health and disability system in Aotearoa New Zealand and their outlined levels of success.

## 3. Results

### 3.1. Literature Search and Review Process

**Figure 1:**
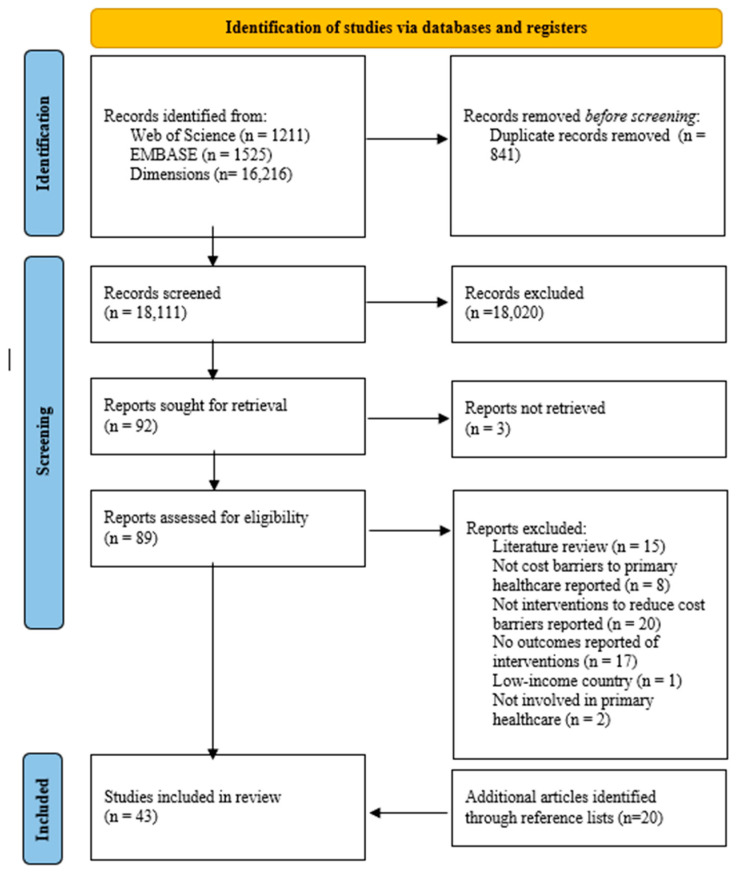
Search and screening flowchart: PRISMA (Preferred Reporting Items for Systematic Reviews and Meta-Analysis) summarizing the identified literature focused on interventions to reduce cost barriers to primary healthcare in high-income countries, 2000–2022.

Based on the publications’ titles and abstracts, 89 articles were retrieved for full-text review from 18,111 potentially relevant citations (Figure 1). The retrieved full-text articles were assessed against the eligibility criteria, and 60 were excluded. The reference lists of eligible full-text articles were scanned for relevant publications, and 20 studies were further included. Following a comprehensive three-phase screening, six publications were excluded due to insufficient information, and one study was a nondisclosed literature review. This brought the total to 43 included articles in this review.

### 3.2. Characteristics of Included Publications

The characteristics of the included articles are presented in Table 3, Table 4, Table 5, Table 6, Table 7 and Table 8. Nearly half (48.8%) of all articles were published between 2015 and 2019. Thirty-one (72.1%) publications were located in the United States, followed by five (11.6%) in Canada and two (4.7%) each in Ireland and Japan. One study (2.3%) was based in each of Aotearoa New Zealand, Australia, and Sweden. Most interventions were implemented at the practice level, including the implementation of nonprofit organizations and community programs (30.2%), alternative payment methods (14.0%), total removal of out-of-pocket costs (11.6%), and additional workforce (4.7%). Other system-level interventions included the ACA in the United States (32.6%) and reforms or initiatives in Sweden and Canada (7.0%). Some interventions targeted specific population groups, such as low-income earners (federal poverty level [FPL] < 200%) (10.9%), individuals with preexisting health conditions (10.9%), children aged under 12 years (6.5%), or older people aged above 65 years (8.8%). A few interventions targeted two population groups concurrently (e.g., low-income earners and those with preexisting health conditions). More than half of the outcomes reported were changes in healthcare utilization (58.1%), followed by cost savings (18.6%), accessibility (14.0%), and medication adherence (9.3%).

### 3.3. Quality of the Evidence

The results of the CASP checklists are summarized in Supplementary Tables S1–S4. Most studies were considered at low risk of bias and were deemed to satisfy the quality assessment. No studies were excluded based on the level of quality appraisal. The results from a few articles were included but interpreted cautiously.

### 3.4. Interventions to Reduce Cost Barriers to Primary Healthcare Services

#### 3.4.1. Total Removal of Out-of-Pocket Costs

All five publications that implemented some form of out-of-pocket removal reported increased healthcare utilization and/or adherence to appropriately prescribed medicines [17,18,19,20,21]. Most studies targeted children under 12 [17,19,20] or older persons above 65 years [18]. Two studies employed interventions in the form of total medical cards [19] and value-based insurance designs [20]. Other articles implemented the complete removal of user charges [17,18,21].

#### 3.4.2. Additional Workforce

Two publications investigated the effect of increasing the workforce to reduce cost barriers experienced by patients in primary healthcare services. One investigation reported that Medicare costs per patient for practices that did not employ nurse practitioners are USD 445 higher than for healthcare services that did employ nurse practitioners [22]. However, the study’s findings did not reach conventional levels of statistical significance. The other study found intervention subjects were more likely to have primary care contact when paired with a health promotion advocate than those who were not paired (51.2% vs. 13.8%, *p* < 0.001) [23]. However, the study appraisal of this investigation was included in this review with caution.

#### 3.4.3. Alternative Payment Methods

Six articles assessed alternative payment methods as an intervention to combat cost barriers to healthcare services. Two investigations reported no significant difference in health utilization and accessibility between intervention and comparison groups [24,25]. One study targeted low-income earners (<182% FPL) and found that insured enrollees were less likely to delay care due to appointment costs than uninsured enrollees (21.3% vs. 78.5%) [26]. Another study targeted older people (aged 64 to 75 years) and reported that out-of-pocket spending was significantly lower than those who were non-eligible for the intervention, aged below 64 years [27]. One investigation examined the reduction of copayments on pharmaceutical products and found that a lower copayment increased medication adherence [28]. Contrastingly, one study found an increase in pharmaceutical copayments was related to decreased medication possession ratios and increased rates of product discontinuation [29].

#### 3.4.4. Reforms and Initiatives

Two out of three investigations reported small or unimproved outcomes. One study reported that the transformation from a needs-weighted capitation formula with age- and area-specific proxies compared to a fee-for-service formula increased doctor visits but observed smaller increases in visits in those with more significant healthcare needs, such as men living in disadvantaged areas [30]. Another investigation reported minimal increases in primary healthcare visits and no substantial changes in average expenditures than the comparison clinics [31]. One analysis investigated a “Mandatory Universal Drug Prescription” in Quebec, Canada, and found pharmaceutical use increased by thirteen per cent relative to other provinces [32].

#### 3.4.5. The Affordable Care Act Expansions

Fourteen studies investigated the impact of the U.S. ACA on mitigating cost barriers to healthcare services [33,34,35,36,37,38,39,40,41,42,43,44,45,46]. Two investigations examined the effects of Medicare’s Part D Prescription Benefit, which targeted those with low incomes and the elderly aged above 65 years [33,34]. Both studies found the benefit of reduced medication expenditures and increased pharmaceutical use. One article reported an increase in out-of-pocket spending among those in the subsidized marketplace coverage (ACA) compared to employer-sponsored insurance (adjusted difference in difference [aDiD]: 9.7 percentage points) [35]. One investigation found that healthcare users were less likely to report barriers to accessing healthcare services after the ACA implementation than in the prior year [36]. Another publication used a stock-and-flow projection model and reported access to healthcare services would improve by about 20.0% under the expansion. However, subsequently, it also reported the availability of physician appointments would decrease by 13.0 to 19.0% [37]. Six of eight articles reported increased physician visits and screening uptake [38,39,40,41,42,43]. Of the two studies that did not, one investigation reported visit rates to primary physicians declined more for dual beneficiaries (who received both Medicaid and Medicare benefits) after the implementation of the ACA than the year before implementation (DiD: −0.37 [95%CI: −0.43 to 0.32] visits per 100 beneficiaries in 2013 to 2014 vs. 2012, *p* < 0.001) [44]. Another investigation reported mixed findings in healthcare utilization between patient populations. Overall, rates of primary healthcare visits did not increase from pre- to post-ACA in the expansion and non-expansion states for diabetic and nondiabetic patients. However, among patients with prediabetes, primary healthcare visit rates in the expansion states increased by 14 per cent (RR: 1.14; 95%CI: 1.08 to 1.19) [45]. One study reported that those who gained and/or maintained Medicaid expansion insurance displayed long-term primary healthcare use patterns similar to those continuously insured [46].

#### 3.4.6. Implementation of Not-for-Profit Organizations and Community-Based Programs

Three studies reported reduced out-of-pocket charges or improved organizational cost savings after introducing some form of a not-for-profit entity. This included interventions such as a clinic operated by nurses [47], community-governed organizations [48], and alternative models of primary healthcare [49]. One study that investigated the impact of a “Community-based Outreach Agency” that employed a multitouch outreach model to foster long-term access to healthcare reported that low-income Latino patients were 60.0% less likely to experience a barrier in accessing primary healthcare, including not being able to afford to pay for a consultation (aOR = 0.4; 95%CI: 0.30 to 0.70) [50]. However, this investigation was outlined and included with caution. Three studies reported that access to services and healthcare utilization improved when financial viability was underpinned by capitation-style funding or enhanced fee-for-service models that included payment incentives and were not entirely reliant on user charges [51,52,53]. Three investigations that reported some form of community healthcare provider found increased visitation and appointment rates compared to the non-exposure group [54,55,56]. One study that investigated Quebec’s Family Medicine Group, a team-based model of care that remained fee-for-service but with additional fixed payments and afterhour availability, found rates of general practitioner visits decreased by 0.45 (*p* < 0.001), and thus, lowered out-of-pocket spending by CAD 11.00 per patient, per year. The investigation further reported finding no evidence that the reduced rate of physician visits was driven by substituting fewer, longer consultations for shorter ones [57]. One article that investigated the effects of a “Case Management Community Health Program”, which involved developing a preventive care regimen tailored to the specific needs of a patient, reported that program participation was statistically associated with an increase in primary healthcare visits from an average of 4.13 to 10.82 (*p* < 0.001) [58]. One investigation that offered enhanced fee-for-service capitation payments through a “Primary healthcare Case Management Program and Performance Incentives” for professionals reported a decline in avoidable hospitalizations and emergency department visits by 16.8% and 5%, respectively [59].

### 3.5. Subgroup Analysis of High-Income Countries with Capitation Formulae

Nine publications (20.9%) were based on high-income countries that employed capitation as a primary funding arrangement for healthcare providers. Over half of the articles (55.6%) involved the “implementation of not-for-profit organizations or community programs” followed by the “removal of out-of-pocket costs” (33.3%), and “initiatives or reforms” (22.2%) from Canada and Sweden. Most outcomes reported comprised healthcare utilization (33.3%), followed by equal parts medication adherence and healthcare accessibility (22.2%), and one study reported on cost savings (11.1%). All publications that removed out-of-pocket costs reported favourable results, with increased physician visits and medication adherence [19,21,59]. Studies that investigated the impact of initiatives or reforms provided mixed results. One study reported no evidence that a reform benefitted those with more significant healthcare needs [30]. Another study reported a 13.0% difference in pharmaceutical use between Quebec and the rest of Canada when a Mandatory Universal Prescription Drug Program was introduced in Quebec [32]. All studies investigating some form of a not-for-profit organization reported reduced out-of-pocket charges and increased physician visits than for-profit organizations [48,57]. A variation of primary healthcare models reported that access to healthcare services, screening uptake, and out-of-pocket costs were improved when financial viability was underpinned by capitation rather than total reliance on fee-for-service models [49,52,53].

**Table 1:** The search strategy of the Web of Science and EMBASE databases.

Database	Search Terms	No. of Articles Retrieved	Limits
Medline Web of Science	(“polic*” OR “strateg*” OR “evaluat*” OR “protocol*” OR “initiat*”) AND (“cost barrier” OR “financ* barrier” OR “financ* challeng* OR “out-of-pocket*” OR “patient cost” OR “user cost” OR “health cost” OR “fee-for-service” OR “health service fee” OR “fee and charg*” OR “affordable” OR “user charg*” OR “patient charg*” OR “health charg*”) AND (“primary health*” OR “community health provid*” OR “community health*” OR “community doctor” OR “community physician” OR “community pract*” OR “community medicine” OR “general health*” OR “general doctor” OR “general physician” OR “general pract*” OR “Māori health*” OR “Māori doctor” OR “Māori physician” OR “Māori pract*” OR “Māori medicine” OR “family doctor” OR “family health” OR “family pract*” OR “family physician”) NOT (“low-income countr*” OR “middle-income countr*”)	1211	Language (English-only publications). Exclusion of review articles (systematic literature, narrative and scoping reviews;book chapters were also excluded). Date: 2000 to 2022.
EMBASE		1525	

**Table 2:** The search strategy of the Dimensions database.

Database	Search Terms	No. of Articles Retrieved	Limits
Dimensions	(“polic*” OR “strateg*” OR “evaluat*” OR “protocol*” OR “initiat*”) AND (“cost barrier” OR “financ* barrier” OR “financ* challeng* OR “out-of-pocket*” OR “patient cost” OR “user cost” OR “health cost” OR “fee-for-service” OR “health service fee” OR “fee and charg*” OR “affordable” OR “user charg*” OR “patient charg*” OR “health charg*”) AND (“primary health*” OR “community health provid*” OR “community health*” OR “community doctor” OR “community physician” OR “community pract*” OR “community medicine” OR “general health*” OR “general doctor” OR “general physician” OR “general pract*” OR “Māori health*” OR “Māori doctor” OR “Māori physician” OR “Māori pract*” OR “Māori medicine” OR “family doctor” OR “family health” OR “family pract*” OR “family physician”) NOT (“low-income countr*” OR “middle-income countr*”)	16,216	Language (English-only publications). Date: 2000 to 2022. High-income countries (Andorra OR Antigua and Barbuda OR Aruba OR Australia OR Austria OR Bahamas OR Bahrain OR Barbados OR Belgium OR Bermuda OR British Virgin Island OR Brunei OR Canada OR Cayman Islands OR Chile OR Croatia OR Curacao OR Cyprus OR Czechia OR Denmark OR Estonia OR Faroe Polynesia OR Germany OR Gibraltar OR Greece OR Greenland OR Hungary OR Iceland OR Ireland OR Isle of Man OR Israel OR Italy OR Japan OR South Korea OR North Korea OR Kuwait OR Latvia OR Liechtenstein OR Lithuania OR Luxembourg OR Malta OR Monaco OR Nauru OR Netherlands OR New Caledonia OR New Zealand OR Norway OR Oman OR Poland OR Portugal OR Qatar OR San Marino OR Saudi Arabia OR Seychelles OR Singapore)

**Table 3:** Study characteristics of included publications grouped by “total removal of out-of-pocket costs”.

Author	Country	Population (n)	Study Design	Intervention (Comparator)	Outcome	Relevant Findings	Limitations	Quality Assessment
McDonnell et al., 2022 [17]	Ireland	General practices (16) and children patients (est. 95,000)	Cohort	Healthcare is free for children under six years (charge for those over six years)	Healthcare utilization	In the two years of the policy’s effect, an additional 3.6 (21%) visits per month per practice were made for every single year of age under six compared to those over the age of six.	Data limitations. Researchers indicated findings may not be nationally representative.	SAT
Murayama et al., 2021 [18]	Japan	National Health Insurance (NHI) beneficiaries aged 40 to 70 years (131, 295)	Cohort	Out-of-pocket cost removal for NHI beneficiaries (before intervention)	Healthcare utilization	Beneficiaries were more likely to receive health checks postintervention than the year prior (odds ratio [ORs] = 1.18; 95% confidence interval [95%CI]: 1.16 to 1.20).	There is a possibility of confounding variables. There is no control group. There is too little follow-up to monitor the sustainability of interventions. Researchers indicated restricted generalizability of findings.	SAT
Nolan and Layte, 2017 [19]	Ireland	Infants aged nine months (9361) and children aged nine years (7163)	Cohort	Gaining a full medical or visit general practice card (user fees)	Healthcare utilization	Gaining a full medical or visit card was associated with a 25% and a 63% increase in number of visits per annum for infants and children, respectively.	A small proportion of the population, therefore, has the possibility of limited statistical power. Intervention effects are noted to be measured in the medium to long term.	SAT
Sepulveda et al., 2016 [20]	United States	Children from a value-based insurance design (25,950)	Cohort	Value-based insurance design including zero out-of-pocket costs for primary healthcare (fee-for-service)	Healthcare utilization	Zero out-of-pocket costs for primary healthcare were associated with an additional 32 visits to a physician per 100 children (95%CI: 27.6 to 36.4, *p* < 0.01) than the control group.	The possibility of confounding variables. Researchers indicated restricted generalizability of findings.	SAT
Persaud et al., 2021 [21]	Canada	Primary care patients not taking medicines due to cost between 1 June 2016 and 28 April 2017 (786)	Randomized controlled trial (RCT)	Free access to 128 essential medicines (usual access to medicines that could involve copayments)	Medication adherence	Adherence to all medicines was 38.7% in the intervention versus 28.6% in the control (absolute difference = 10.1%; 95%CI: 3.3 to 16.9, *p* = 0.004).	Group allocation is not blinded. Medicine adherence is ascertained using patient records. Baseline medicine adherence was not measured.	SAT

Abbreviation: NHI: National Health Insurance; FFS: fee-for-service; RCT: randomized controlled trial; OR: odds ratio; 95%CI: 95% confidence interval. SAT: The quality appraisal of the study reached satisfactory levels as determined by the CASP checklist.

**Table 4:** Study characteristics of included publications grouped by “additional workforce”.

Author	Country	Population (n)	Study Design	Intervention (Comparator)	Outcome	Relevant Findings	Limitations	Quality Assessment
Horwitz et al., 2005 [23]	United States	Uninsured patients at least 18 years old and who did not have a regular primary healthcare provider (230)	RCT	Intensive case management program with a health promotion advocate (ysual care)	Healthcare utilization	Intervention subjects were more likely to have primary healthcare contact than the comparison group (51.2% vs. 13.8%, *p* < 0.0001).	Researchers indicated restricted generalizability of findings. A small proportion of the population, therefore, has the possibility of limited statistical power.	CAUT
Krawelski et al., 2015 [22]	United States	Primary care practice (85) matched with Medicare patients (315,000)	Economic evaluation	Practices with nurse practitioners (NPs) (practices with no NPs)	Cost savings	Medicare costs per patient were USD 445 higher for practices that do not employ NPs compared to those that do employ NPs.	The possibility of confounding variables. Not able to link causality.	SAT

Abbreviations: NPs: nurse practitioners; CAUT: no studies were excluded on the basis of the level of quality appraisal. However, as determined the CASP checklist, the article was included with caution.

**Table 5:** Study characteristics of included publications grouped by “alternative payment methods”.

Author	Country	Population (n)	Study Design	Intervention (Comparator)	Outcome	Relevant Findings	Limitations	Quality Assessment
Heintzman et al., 2019 [25]	United States	All patients who received care at study clinics from 1 July 2012 to 28 February 2015 (18 practices)	Cohort	Primary clinics enrolled in alternative payment methods [APM] (comparison clinics [non-APM], FFS)	Healthcare utilization	APM clinics had a lower rate of total office encounters and new patient visits than non-APM clinics. (Relative rates [RRs] = 0.97; 95%CI: 0.89 to 1.05 and RR = 0.81; 95%CI: 0.53 to 1.23, respectively.)	The possibility of results subject to selection bias. The possibility of confounding variables. The study was not randomized.	SAT
Alcala et al., 2018 [24]	United States	Adults aged 18 to 64 years insured through Medicaid, privately purchased insurance or employer-sponsored coverage (20,258)	Cross-sectional	Private individual market insurance plans [on- and off-exchange] and Medicaid (employer-sponsored insurance)	Accessibility	Medicaid individuals and those who purchased private coverage on- and off-exchange were more likely to not be accepted as new patients in the past 12 months (Medicaid: ORs = 3.13; 95%CI: 2.04 to 4.79; private coverage on-exchange: ORs = 2.47; 95%CI: 1.39 to 4.38; and private coverage off-exchange: ORs = 1.92; 95%CI: 1.07 to 3.46, respectively).	Causality cannot be determined. Researchers indicated restricted generalizability of findings.	SAT
Feinglass et al., 2014 [26]	United States	Low-income [182% FPL] uninsured residents of DuPage County (293)	Cross-sectional	Established Access DuPage (AD) enrollees (New AD enrollees asked about the previous year when uninsured)	Accessibility	Those uninsured reported a higher likelihood of delaying medical care due to the cost of a visit (78.5% vs. 21.3%, *p* < 0.0001) than established enrollees.	The possibility of confounding variables. No control group was present. Unable to assess changes over time.	SAT
Landsman et al., 2005 [29]	United States	Managed care enrollees (1,630,000)	Cohort	Three-tier pharmacy benefit with varying levels of copayments (two-tier benefit scheme, with copayment levels unchanged)	Medication adherence	The increase in tiers and copayments decreased medication possession ratios and increased switching to lower-price alternatives and product discontinuation rates.	Discontinuation rates may have been overestimated. Elasticizes may have been overestimated.	SAT
Maciejewski et al., 2010 [28]	United States	Self-insured employers representing enrollees with preexisting conditions (32,259 employers, 1,385,391 enrollees)	RCT	Value-based insurance design program that included eliminating generic medication copayments and reducing copayments for brand-name medications (employers not in the program)	Medication adherence	Medication adherence improved by two to four percentage points within six therapeutic classes in the program’s first year relative to the comparison group.	Assumptions made in data measurements. Unable to assess changes over time.	SAT
Nishi et al., 2012 [27]	Japan	Individuals aged between 64 and 75 years (10,293)	Cross-sectional	Reduced copayments in cost-sharing methods from 30% to 10% after turning 70 years with an annual taxable income under USD 12,000 (those younger than 70 years)	Cost savings	Out-of-pocket spending was significantly lower for eligible adults (*p* < 0.001) than for those younger than 70 years.	The possibility of results subject to selection bias. The study cannot infer causation. Lack of data; unable to identify individuals with treatable chronic conditions.	SAT

Abbreviations: APM: alternative payment methods; AD: Access DuPage program; RR: relative rates.

**Table 6:** Study characteristics of included publications grouped by “reforms and initiatives”.

Author	Country	Population (n)	Study Design	Intervention (Comparator)	Outcome	Relevant Findings	Limitations	Quality Assessment
Agerholm et al., 2015 [30]	Sweden	Randomly chosen individuals in Stockholm County above 18 years (65,474)	Cross-sectional	Reimbursement system change and choice reform to FFS (weighted capitation with age- and area-specific proxies)	Healthcare utilization	The mean number of physician visits increased for all groups from pre- to post-intervention (56% vs. 65%, a relative increase of 1.17). However, men living in disadvantaged areas had a significantly smaller increase than their reference groups.	Only two time points were measured. Nonrespondents were stated to be overrepresented in social and economically disadvantaged groups. There is the possibility of confounding variables.	SAT
Peikes et al., 2018 [31]	United States	General practices (1005)	Cohort	Comprehensive Primary Care Imitative (CPC) for Medicare and Medicaid services (matched comparison practices)	Healthcare utilization	There were smaller increases for CPC services than comparison clinics in primary care visits in all settings. Relative to average expenditures in comparison clinics, those in CPC practices increased USD 9.00 less without care management fees and USD 6.00 more with fees.	Practices were not randomly assigned. Data measurements were limited. Researchers indicated restricted generalizability of findings.	SAT
Wang et al., 2015 [32]	Canada	One member of a household between the ages of 12 and 56 in 1994/5 (10,653)	Cross-sectional	Mandatory Universal Prescription Drug (UPD) insurance program in Quebec (rest of Canada)	Medication use/adherence	The UPD program led to a 13% increase in medication in the previous month for those in Quebec than those in other provinces. The UPD was not associated with being more likely to visit a doctor in the previous month.	None stated.	SAT

Abbreviations: CPC: Comprehensive Primary Care Initiative; UPD: Universal Prescription Drug Program.

**Table 7:** Study characteristics of included publications grouped by “the Affordable Care Act expansions”.

Author	Country	Population (n)	Study Design	Intervention (Comparator)	Outcome	Relevant Findings	Limitations	Quality Assessment
Bailey et al., 2022 [38]	United States	Cancer survivors (2917)	Cohort	Medicaid expansion	Healthcare utilization	Cancer survivors in expansion states had higher odds of having greater than or equal to six visits to primary healthcare than cancer survivors in the non-expansion states (OR = 1.82; 95%CI: 1.22, 2.73).	Unable to measure replacement therapies or medications. The possibility of confounding variables. Some cancer survivors not identified.	SAT
DeVoe et al., 2015 [40]	United States	Adults aged 19 to 64 years in the Oregon Experiment (34,849)	RCT	Medicaid (not selected to apply for Medicaid)	Healthcare utilization	Those who received Medicaid had significantly more primary healthcare visits than those who did not receive Medicaid, with 81 additional visits per 1000 Medicaid-covered patients per month (adjusted rate ratio [aRR] = 1.39; 95%CI: 1.16 to 1.66).	Limited data measurements. Researchers indicate restricted generalizability of findings. The models used assume that instruments are not correlated with the outcomes.	SAT
Fung et al., 2021 [44]	United States	Dual-eligible [Medicare and Medicaid] and non-dual-eligible beneficiaries [Medicare with low income whose fees did not change] in 2012 (3,052,044)	Cross-sectional	Medicaid fees bump in 2013 to 2014 and Bump extension in 2015 to 2016 (prebump in 2012)	Healthcare utilization	Visit rates with primary care physicians declined for dual-eligible benefits compared to non-dual-eligible benefits across time periods (difference in difference [DiD] = −0.37 visits per 100 beneficiaries; 95%CI: −0.43, to −0.32 in 2013 to 2014 vs. 2012, *p* < 0.001, and DiD = −0.62 visits per 100 beneficiaries; 95%CI: −0.68 to −0.56 in 2015 to 2016 vs. 2012, *p* < 0.001).	The possibility of confounding variables. Limited data. Unable to examine changes in practitioners’ panel compositions.	SAT
Hatch et al., 2016 [46]	United States	Low-income, uninsured adults aged 19 to 64 years (8069)	Cohort	Gained, maintained, or lost Medicaid expansion (continuously insured and uninsured)	Healthcare utilization	Those who gained and maintained Medicaid expansion insurance showed long-term primary healthcare utilization patterns similar to those continuously insured.	Researchers indicate restricted generalizability of findings. Not sufficient data for long-term changes. The possibility of confounding variables. Researchers did not measure the duration and reason for lost insurance.	SAT
Heintzman et al., 2017 [41]	United States	Low-income Latino adults aged 21 to 79 years with at least a primary healthcare visit from 2009 to 2013	Cross-sectional	One year post-ACA in 2014 (pre-ACA from 2009 to 2013)	Healthcare utilization	Among 5926 uninsured patients, 81.2% gained insurance postintervention. The greatest impact was seen among Hispanic/Latino patients, with an absolute change of −56% in the uninsured rate (pre-ACA 65.3% vs. post-ACA 13.7%).	Researchers indicate restricted generalizability of findings. Limited data.	SAT
Hoopes et al., 2016 [42]	United States	Community health centers (CHCs) and adult patients aged 19 to 64 years (219 CHCs and 401,988 patients)	Cross-sectional	Post-expansion in expansion states and non-expansion states (pre-expansion in expansion states and non-expansion states)	Healthcare utilization	In the group of expansion states, visits to CHCs postexpansion for a new patient increased by 14% (RR = 1.14; 95%CI: 1.14, to 1.73) and visits to primary healthcare increased by 6% (RR = 1.06; 95%CI: 1.02 to 1.10) compared to pre-expansion.	Researchers indicate restricted representation of all CHC, states, or expansion status groups. This analysis does not assess patient-level insurance or changes in the patient panel. The possibility of confounding variables.	SAT
Huguet et al., 2018 [45]	United States	Nonpregnant patients aged 19 to 64 years with greater than one ambulatory visit between 1 January 2012 and 31 December 2015 from CHCs (198 CHCs and 872,378 patients)	Cross-sectional	Post-ACA (pre-ACA) among a cohort of patients with diabetes, prediabetes, and no diabetes	Healthcare utilization	Primary healthcare visit rates did not increase for diabetes and no-diabetes cohorts from pre- to post-ACA in expansion states and non-expansion states. However, among prediabetes, primary healthcare visits increased significantly (RR = 1.14; 95%CI: 1.09 to 1.19).	Researchers indicated findings are not representative. The possibility of confounding variables.	SAT
Wherry and Miller, 2016 [43]	United States	Low-income adults aged 19 to 64 years [138% FPL] (40,427)	Cross-sectional	Expansion states (non-expansion states)	Healthcare utilization	Visits to the general doctor in the last 12 months increased significantly in the expansion states compared to the non-expansion states (6.6 percentage points; 95%CI: 1.13 to 12.0).	The possibility of confounding variables. The possibility of self-reported data and recall bias. Limited timeframe to measure the sustainability of the intervention. Examining multiple outcomes increased the probability of estimates found by chance.	SAT
Cohen et al., 2012 [39]	United States	Medicaid Advantage enrollees with diabetes (3164)	Cohort	Medicare Advantage Chronic Condition Special Needs Plans [SNPs] (FFS)	Healthcare utilization	The percentage difference in utilization rates for physician office visits among all patients with diabetes was five percentage points higher for Medicare Advantage C-SNP versus FFS. The percentage difference for nonwhite diabetes patients was 14 percentage points higher for Medicare Advantage C-SNP versus FFS in physician office visits.	Limited statistical prevision. Not causation analysis. Researchers used datasets from different years.	SAT
Bustamante and Chen, 2018 [36]	United States	Noninstitutionalized population aged 18 to 64 years (91,680)	Cross-sectional	Affordable Care Act insurance mandate (before implementation in 2011)	Accessibility	Post-ACA, adults reported lower odds of having trouble finding a doctor (in 2012: OR = 0.88; 95%CI: 78 to 1.00, *p* < 0.04; in 2013: OR = 0.80; 95%CI: 0.71 to 0.90, *p* < 0.001, and in 2014: OR = 0.80; 95%CI: 0.71 to 0.90, *p* < 0.01) and healthcare providers not accepting healthcare insurance (OR = 0.80; 95%CI: 0.70 to 0.91, *p* < 0.001 in 2013 and OR = 0.81; 95%CI: 0.73 to 0.91, *p* < 0.001 in 2014) compared to pre-ACA.	There is a possibility of bias due to self-reported data. The analysis distinguishes among only four regions, limiting generalizability.	SAT
Gentili et al., 2016 [37]	United States	Adults aged 19 to 64 years in Georgia excluding Medicare population (not given)	Projection model: stock and flow	Affordable Care Act (business-as-usual)	Accessibility	It was projected that the ACA implementation would have a positive impact on accessibility to healthcare appointments by about 20% but a negative effect on the availability of appointments (13% to 19% decrease).	Reliance on model assumptions. Need estimates were based on utilization ratios. Limited data.	SAT
Goldman et al., 2018 [35]	United States	Adults aged 18 to 63 years (9653)	Cross-sectional	Subsided marketplace coverage [ACA-affected cohort] (employer-sponsored insurance [pre-ACA])	Cost savings	Out-of-pocket expenses increased among adults with any expenditures in the intervention versus control (aDiD = 9.7 percentage points).	A small proportion of the population; therefore, has the possibility of limited statistical power. Data limitations. Short timeframe.	SAT
Yin et al., 2008 [33]	United States	Five per cent of pharmacy customers filled at least one prescription between 2005 and 2006 (177,311)	Cross-sectional	Persons aged 66 to 79 years eligible for Part D Prescription Benefit [PDPB] (non-eligible for enrolment those aged 60 to 63 years)	Cost savings	Expenditures on medication were reduced by USD 5.20 per month (31.1%, *p* = 0.003) and increased medicine use by 3.7 pill-days (95.9%, *p* < 0.001) for those aged 66 to 79 years after the implementation of the PDBP.	The possibility of confounding variables. The approach assumes that the absence of a prescription claim represents no utilization rather than missing data. Data limitations.	SAT
Zhang et al., 2010 [34]	United States	Four per cent of individuals enrolled in Medicare Advantage Plans (34,176)	Cross-sectional	Medicare drug benefit, Part D [USD 150 or USD 350 quarterly caps] (stable drug coverage from 2004 to 2007)	Cost savings	Part D reduced out-of-pocket spending by 13.4% (95%CI: −17.1 to −9.1) among those without prior coverage and 15.9% among those with USD 150 (95%CI: −19.1 to −12.8) quarterly caps and remained the same in the USD 350 capped group relative to comparison.	Researchers indicate limited generalizability of findings. Possibility of measurement error.	SAT

Abbreviations: aRR: adjusted rate ratio; RR: rate ratio; aDiD: adjusted difference in difference; PDPB: Part D Prescription Benefit.

**Table 8:** Study characteristics of included publications grouped by “implementation of not-for-profit organizations and community programs.”

Author	Country	Population (n)	Study Design	Intervention (Comparator)	Outcome	Relevant Findings	Limitations	Quality Assessment
Bradley et al., 2012 [54]	United States	Uninsured low-income adults without children enrolled in a community-based primary care program (26,284)	Cross-sectional	Community-based primary care provider (patients newly enrolled for less than one year)	Healthcare utilization	By the third year of enrolment, the average number of primary healthcare visits increased to 1.60 per annum, and emergency department [ED] visits fell to 0.74 per year.	Data restrictions for in-depth analysis. Difficult to infer causality.	SAT
Burton et al., 2002 [51]	United States	Elder Health patients matched with groups of dually eligible older individuals aged 65 and above (401)	Qualitative	Private, for-profit healthcare organization [Elder Health] that combines Medicare and Medicaid capitation payments at the healthcare provider level (FFS)	Healthcare utilization	By the one-year follow-up, a greater proportion of Elder Health patients remained highly satisfied with access to care compared to FFS patients (22.5% vs. 7.1%, *p* < 0.001). Elder Health patients had a higher combined rate of physician and nurse visits than the FFS comparison group (14.2 vs. 11.5).	A small proportion of the population; therefore, has the possibility of limited statistical power. Researchers indicate limited generalizability of findings. Not randomized, therefore, cannot infer causality. Limited data.	SAT
Glendenning-Napoli et al., 2012 [58]	United States	Those without medical insurance aged 18 to 65 years with chronic diseases (83)	Cross-sectional	Community Health Program (CHP) that includes a registered nurse developing a preventive care regimen tailored to the specific needs of the patient (pre-CHP)	Healthcare utilization	Participation in CHP was associated with a statistically concomitant increase in primary healthcare visits from a mean of 4.13 to 10.82 (*p* < 0.0001). The total number of primary healthcare visits increased by 162% from 343 pre-CHP to 898 post-CHP.	The possibility of selection bias. Researchers indicate limited generalizability of findings. Researchers failed to factor in the administrative costs of intervention. Cost savings may have been overestimated.	SAT
McMorrow and Zuckerman, 2014 [55]	United States	Low-income <200% [FPL] adults aged 19 to 64 years (not given)	Cross-sectional	A USD 10 increase in funding to CHC per poor person for low-income adults (business-as-usual)	Healthcare utilization	The funding growth positively and significantly impacted the probability of having an office visit for all low-income adults. A similar pattern emerged for the probability of a general doctor visit but no other significant measures of access to care.	Researchers indicate limited generalizability of findings. A small proportion of the population, therefore, has the possibility of limited statistical power. Measurement error in estimates of market-level center funding.	SAT
Phillips et al., 2014 [59]	United States	Illinois Medicaid members (8,634,604)	Cross-sectional	Illinois Health Connect [IHC], a case management program for Medicaid that offered enhanced fee-for-service capitation payments and performance incentives and Your Healthcare Plus [YHP], a complementary disease management program (before implementation)	Healthcare utilization	Avoidable hospitalizations fell by 16.8% for YHP, and bed days fell by 15.6% for IHC. Emergency department visits declined 5% for IHC and 4.6% for YHP by 2010.	Administrative data limitations. The possibility of unmeasured confounders. Cannot declare causal relationships. The possibility of publication bias.	SAT
Richards et al., 2014 [56]	United States	Telephone calls posing as patients (10,904 calls)	RCT	Federally Qualified Health Centers (FQHC) (non-FQHC)	Healthcare utilization	Medicaid appointment rates at FQHCs are 22 percentage points higher than other primary healthcare clinics, irrespective of the caller, clinic, and other area variables. Nearly 70% of FQHCs provide low-cost (<USD 100) visits to self-pay patients compared to 40% of non-FQHCs.	Researchers indicate that findings are not representative. The study focused on one type of dimension of access and used a single marker of care quality among outcome measures.	SAT
Strumpf et al., 2017 [57]	Canada	Chronically ill patients who registered with a general practitioner between January 2003 and 2005 (579,541)	Cross-sectional	Family Medicine Groups [FMGs] include extended hours and multidisciplinary teams, but they maintain the same remuneration FFS scheme (non-FMGs)	Healthcare utilization	The number of general practitioner visits for FMGs fell by 0.45 visits per patient per year, and spending was reduced by CAD 11.00 per patient per year. This finding is consistent with the authors’ hypothesis regarding improved quality and continuity of care.	None stated.	SAT
Lofters et al., 2018 [52]	Canada	Ontario family physicians (not given)	Cross-sectional	Transition to Enhanced FFS, including incentives and bonuses (transition from traditional FFS)	Healthcare utilization	The transition was associated with increased cancer screening uptake for long-term residents, immigrants, and people in the lowest and highest income quintiles. However, it appeared less beneficial for more disadvantaged groups, including foreign-born and low-income patients, as it was associated with widening screening inequities.	Underestimation in cervical screening. Heterogeneity among practices unaccounted for. Researchers indicate that findings are not generalizable. The method of enrolments differed at time points. Databases not 100% sensitive to migration of provinces.	SAT
Saluja et al., 2012 [50]	United States	Low-income Latinos through Los Angeles County in 2019 (306)	RCT	Enrolled in the Children’s Health Outreach Initiative [CHOI] for 11 to 13 months (those newly enrolled in the program)	Accessibility	Those in the intervention group were less likely to experience barriers to primary healthcare, including not being able to afford to pay for a visit (aOR = 0.4; 95%CI: 0.3 to 0.7), than in the comparison group.	The possibility of unmeasured confounders. Purposive selection of participants. Cannot draw causation. The possibility of recall biases based on self-reported measures.	SAT
Ward et al., 2018 [53]	Australia	Colocated and multidisciplinary primary healthcare services (6 practices)	Qualitative	Capitation models of primary healthcare (traditional FFS)	Accessibility	Access arrangements (including availability and accommodation, affordability, acceptability, appropriateness, and approachability) were improved when financial viability was underpinned by capitation-style funding models and not totally reliant on FFS funding.	Limited sample size. Aboriginal communities not included. Information comes from discussion with stakeholders, not patients.	SAT
Bicki et al., 2013 [47]	United States	Patients of the “CHEER” Clinic (Clinica Esperanza/Hope Clinic Non-Urgent Care Walk-in) (256)	Cross-sectional	Walk-in services run by nurses [CHEER Clinic]; a nurse discusses the plan of care with the patient and determines whether a referral is needed or if the patient is added to the parent clinic’s waiting list to receive follow-up (business-as-usual)	Cost savings	The overall cost savings of the CHEER clinic amounted to USD 760 per patient who would have sought care in the ED. Dividing these savings by the clinic’s operation cost yields a mean return of USD 34 per USD 1 invested.	Researchers indicate the possibility of overestimating findings. Self-reported data, possibility of recall biases, missing data.	SAT
Crampton et al., 2005 [48]	Aotearoa New Zealand	General practitioner respondents (262)	Cross-sectional	Community-governed nonprofit primary healthcare providers (for-profit primary healthcare providers)	Cost savings	A higher percentage of practices with reduced charges were nonprofit providers compared to for-profit providers (61.9% vs. 16.5%, *p* < 0.001). Nonprofit practices waived a higher percentage of charges than for-profit counterparts (38.5% vs. 6.8%, *p* < 0.001).	The possibility of self-reported biases. Nonrespondents.	SAT
Laberge et al., 2017 [49]	Canada	Ten per cent of a random sample who had a valid healthcare card on the 1 April 2012 (1,133,645)	Cross-sectional	Enhanced FFS models, Comprehensive Care Model [CCM], and Family Health Group [FHG] (fee-for-service)	Cost savings	On average, primary healthcare costs for one year were USD 32 lower for CGM and USD 13 lower for FHG patients compared to FFS patients.	Limited data.	

Abbreviations: ED: emergency department; IHC: Illinois Health Connect; YHP: Your Healthcare Plus; FQHC: Federally Qualified Health Centers; CHOI: Children’s Health Outreach Initiative; CCM: Comprehensive Care Model; and FHG: Family Health Group.

## 4. Discussion

To our knowledge, this is the first systematic review of the international literature to examine interventions implemented to reduce cost barriers to primary healthcare in high-income countries. Through this synthesis of evidence, we identified the type of interventions most commonly employed to reduce cost barriers in primary healthcare and which outcomes were most affected. This review followed a rigorous methodology and comprised 43 peer-reviewed articles which covered six broad groups of interventions. In total, most publications presented in this review found statistically significant improvements, whilst a few reported mixed or nonstatistically significant findings in their primary outcomes.

From the synthesis of the literature, several key findings emerged. The most consistent was that the complete removal of out-of-pocket costs was associated with significant improvements in doctor visitation rates and medication possession ratios. Most publications in this intervention group targeted specified populations, such as children and/or older adults. Therefore, the generalizability of the literature findings and, thus, the feasibility of this intervention to other population cohorts remains unclear. Likewise, interventions that lowered out-of-pocket costs through alternative payment methods, such as cost sharing, generally reported improved medication adherence and access to primary healthcare outcomes. Studies that investigated the effects of increasing the healthcare workforce reported an increased uptake of follow-up appointments and lower Medicaid expenditure per person per practice. Health system initiatives generally, but not consistently, were associated with improved access to healthcare services. Of interest, Sweden’s transition from a needs-weighted capitation formula with age- and area-specific proxies to fee-for-service funding arrangements found significantly smaller increases in the number of visits for those with more significant healthcare needs than those who were healthy. This study finding demonstrates that the transition to a healthcare system that relies predominantly on out-of-pocket payments of health users does not benefit those with greater healthcare needs to the same extent as those with lesser needs, exacerbating health inequities and, consequently, worse healthcare outcomes. In contrast, Quebec’s Mandatory Universal Prescription Drug Program provided public medicine coverage for those who do not have private plans and found an increase in medication use and practitioner visits. Most articles published in the United States investigated the impact of the ACA and found, on average, that expansion states showed improved outcomes relative to non-expansion states or found that physician visitation rates were higher postimplementation of the ACA than a year prior.

This review found that capitation-style funding increased access to healthcare services and screening uptakes and reduced out-of-pocket costs for users compared with healthcare providers with complete reliance on fee-for-service funding arrangements. Although some capitation funding arrangements still required out-of-pocket gap payments for healthcare services, this is, comparatively, much lower than fee-for-service arrangements in which users pay for the total care charge. This full charge substantially deters those from seeking healthcare, contributing to a patient’s health status deterioration, and is more likely to significantly impact those who are less financially stable [10,11]. Service-level interventions, such as not-for-profit organizations and community-based programs, reported favourable results in reduced out-of-pocket costs and increased physician visitation rates compared to for-profit organizations. As highlighted in an Aotearoa New Zealand study included in this review [45], not-for-profit organizations and community-based programs were more likely to provide health services at reduced costs or zero charges. This limited or eliminated the financial barrier experienced when accessing healthcare providers and, more often, resulted in an increased rate of physician consultations than for-profit organizations. This review found the total removal of out-of-pocket costs for service users demonstrated increased visits to a physician and improved medication adherence. This finding reflects the simplicity of this intervention and its effects. Entirely removing out-of-pocket costs and, thus, eliminating the financial barrier in accessing primary services will, in return, increase patient healthcare utilization and medicine adherence. However, it is important that access to high-quality primary healthcare is maintained. The provision of free healthcare appointments could lead to increased demand, and without adequate investment in staffing and service funding, this could result in a shortage of physician appointments, creating another barrier to access. For instance, a stock-and-flow project model included in this review found that while the ACA expansion increased healthcare access, it also led to a decrease in the availability of physician appointments [37]. Our review reported the possible effectiveness of increasing the workforce, which led to improved cost savings and healthcare utilization for the patient [22,23]. A hybrid model that combines a variety of interventions, such as the elimination of out-of-pocket costs and the increased utilization of nurse practitioners, may be worth considering to improve access to primary healthcare.

Cost barriers to accessing primary healthcare services ingrain health inequity at the system level. Out-of-pocket costs in healthcare can be crippling to the financial stability of an individual and their household [7,10,11]. More often, those with lower incomes may choose to forgo healthcare, contributing to a deterioration of health status [10,11]. This worsening increases the likelihood of experiencing poverty by reducing one’s ability to work and, consequently, declining wages associated with paid employment [60]. This cycle has been demonstrated globally, as those who cannot improve their financial capacity to afford healthcare due to poor health status fall deeper into poverty-related circumstances [60]. Furthermore, the repercussions of out-of-pocket costs associated with healthcare can transcend to affect other areas of living. For example, as healthcare expenditures increase, this reduces the amount of disposable income available to spend in different areas, such as food, transport, accommodation, or entertainment [60].

The primary healthcare system in Aotearoa New Zealand is largely funded through capitation-based arrangements for general practices, supplemented by user copayments [5]. The shift from fee-for-service towards universal-weighted capitation was introduced through a set of primary healthcare reforms aimed at lowering patient copayments and promoting population health [61,62]. The capitation funding formulae supplied to healthcare providers are based on historical utilization rates of general practices and the age and sex of patients enrolled [63]. Over the past decade, many organizations have raised concern that the formulae fail to consider a community’s level of deprivation and ethnicity and question whether the formulae exacerbate existing inequities or act to address them [61,63,64]. In this review, few studies were based in high-income countries that employed capitation as a primary funding scheme for healthcare providers, similar to Aotearoa New Zealand. Therefore, this limits any generalizability of findings and the implications of interventions if implemented in Aotearoa New Zealand’s health and disability system.

### Strengths and Limitations

As a strength, this literature review explored real-world interventions in high-income countries and identified six interventions to reduce cost barriers to primary healthcare for all populations. Furthermore, this review exclusively analyzed high-income countries that employed capitation as a primary funding source for healthcare providers in a subgroup analysis to investigate cost-reducing methods most complementary to the health and disability system in Aotearoa New Zealand. Finally, this review demonstrates that the complete removal of out-of-pocket costs to healthcare eliminates financial barriers experienced by healthcare users and provides consistent and favourable results. Likewise, reducing out-of-pocket user charges through cost-sharing methods also provides beneficial outcomes, but less consistently.

This review does have limitations that should be considered carefully. First, we relied entirely on electronic databases to search for relevant citations and included only English publications. It is possible other relevant studies could have been eligible for this review if we were to include articles published other than in the English language or used broader forms of search engines, such as grey material and presentation notes. Second, some interventions targeted specific populations, e.g., the complete removal of out-of-pocket costs targeting children or older people. The impact of such interventions on a population level may not be the same as that on specific groups. Third, there is concern about whether increased health utilization is an appropriate measure of improvement in primary healthcare access. Various international investigations have raised awareness that the quantity of healthcare services is not always a substitute for quality [65]. As addressed in our review, it is possible that patients are able to attend more healthcare services after the intervention, but the quality of care received may be reduced, predisposing them to worse patient health outcomes. For example, as healthcare services become more affordable, the demand and uptake of such services from patients increases. To meet this increase in demand, physicians must be able to fit a surplus of consultations in the same number of working hours, often with inadequate funding to do so. Therefore, appointments become shorter, and the relationship between practitioner and client may suffer. To mitigate this effect, it is worth considering a hybrid model that combines a variety of interventions, such as investment in staffing levels and the removal of out-of-pocket costs to improve access to primary healthcare. Lastly, financial barriers to primary healthcare are limited not only to out-of-pocket costs associated with the healthcare service but also to other payments, such as transportation, paying caregivers for dependents, and opportunity costs, such as time off work [5]. Other barriers to primary healthcare can include but are not limited to gender-based and/or cultural constraints, lack of health literacy, and poor infrastructure support [66,67]. Therefore, we emphasize the importance of a multidimensional view of healthcare access, as proposed by Levesque et al. [68]. This framework considers the socioeconomic determinants of individuals to identify, seek, reach, obtain, and engage in primary healthcare services [69].

## 5. Conclusions

Many international efforts have been implemented to reduce cost barriers to primary healthcare in high-income countries, with differing levels of success. In this review, the complete removal or lowering of out-of-pocket costs through direct or alternative payment payments, such as cost sharing, significantly increased healthcare use and medication adherence. Universal healthcare with financial risk protection is a core objective of the Sustainable Development Goals. The persistence of out-of-pocket costs in primary healthcare works against commitments outlined by Member States and disproportionately affects those most vulnerable, predisposing them to catastrophic healthcare expenditures and income poverty. A range of initiatives to improve access to primary healthcare could address these problems to impact health equity and reduce overall health system costs.

## Data Availability

The data presented in this review are available on request by the corresponding author.

## Supplementary Materials

The following supporting information can be downloaded at https://www.mdpi.com/article/10.3390/ijerph21081029/s1, Table S1: Quality Appraisal of Economic Evaluations: CASP Checklist; Table S2: Quality Appraisal of Cohort Studies: CASP Checklist; Table S3: Quality Appraisal of Qualitative Studies: CASP Checklist; Table S4: Quality Appraisal of Randomized Controlled Trials: CASP Checklist.

## Abbreviations

PRISMA: Preferred Reporting Items for Systematic Reviews and Meta-analysis; CASP: Critical Appraisal Skills Program; ACA: Affordable Care Act; FPL: federal poverty level; NHI: National Health Insurance; FFS: fee-for-service; RCT: randomized controlled trial; OR: odds ratio; 95%CI: 95% confidence interval; NPs: nurse practitioners; APM: alternative payment methods; AD: Access DuPage program; RR: relative rates; CPC: Comprehensive Primary Care Initiative; UPD: Universal Prescription Drug program; aRR: adjusted rate ratio; RR: rate ratio; aDiD: adjusted difference in difference; PDPB: Part D Prescription Benefit; ED: emergency department; IHC: Illinois Health Connect; YHP: Your Healthcare Plus; FQHC: Federally Qualified Health Centers; CHOI: Children’s Health Outreach Initiative; CCM: Comprehensive Care Model; FHG: Family Health Group.
