# Supplementary material for: What Interventions Work to Reduce Cost Barriers to Primary Healthcare in High-Income Countries? A Systematic Review
Source: Int J Environ Res Public Health. 2024 Aug 5;21(8):1029. doi: 10.3390/ijerph21081029 (PMC11353906; doi:10.3390/ijerph21081029)
Supplement: Supplementary file 1 [file ijerph-21-01029-s001.zip › ijerph-3136784-supplementary.pdf]

Supplementary Table S1. Quality Appraisal of Economic Evaluation: CASP Checklist

Author (Year)	1	2	3	4	5	6	7*	8	9	10	11	12	Overall Appraisal
Bicki et al., (2022)	✓	✓	✓	✓	✓	✗		✓	✓	✓	✗	✓	SAT
Kralewski et al., (2015)	✓	✓	✓	✓	✗	✗		✗	✗	✓	✗	✓	SAT

*Results of the associated study can be found in the 'Study characteristics tables', see Tables 3 through 8.

Supplementary Table S2. Quality Appraisal of Cohort Studies: CASP Checklist

Author (Year)	1	2	3	4	5a	5b	6a	6b	7*	8	9	10	11	12	Overall Appraisal
Agerholm et al., (2015)	✓	✓	✓	✓	✓	✓	n/a	n/a			✓	✗	✗	✓	SAT
Alcala et al., (2018)	✓	✓	✓	✓	✓	✓	n/a	n/a			✓	✗	✓	✓	SAT
Bailey et al., (2022)	✓	✓	✓	✓	✓	✓	✓	✓			✓	✗	✓	✓	SAT
Bradley et al., (2012)	✓	✓	✓	✓	✓	✓	n/a	n/a			✓	✗	✓	✓	SAT
Cohen et al., (2012)	✓	✓	✓	✓	✓	✓	n/a	n/a			✓	✗	✓	✓	SAT
Crampton et al., (2005)	✓	✓	✓	✓	✓	✓	n/a	n/a			✓	✓	✓	✓	SAT
Feinglass et al., (2014)	✓	✓	✓	✓	✓	✓	n/a	n/a			✓	✗	✓	✓	SAT
Fung et al., (2021)	✓	✓	✓	✓	✓	✓	✓	✓			✓	✗	✓	✓	SAT
Gentili et al., (2016)	✓	✓	✓	✓	✓	✓	n/a	n/a			✓	✗	✓	✓	SAT
Glendenning-Napoli et al., (2012)	✓	✓	✓	✓	✓	✓	n/a	n/a			✓	✗	✓	✓	SAT
Goldman et al., (2018)	✓	✓	✓	✓	✓	✓	✓	✓			✓	✗	✓	✓	SAT
Hatch et al., (2016)	✓	✓	✓	✓	✓	✓	✓	✓			✓	✗	✓	✓	SAT
Heintzman et al., (2017)	✓	✓	✓	✓	✓	✓	✓	✓			✓	✗	✓	✓	SAT
Heintzman et al., (2019)	✓	✓	✓	✓	✓	✓	✓	✓			✓	✗	✓	✓	SAT
Hoopes et al., (2016)	✓	✓	✓	✓	✓	✓	✓	✓			✓	✗	✓	✓	SAT
Hsu et al., (2003)	✓	✓	✗	✓	✗	✗	n/a	n/a			✗	✓	✓	✓	CAUT

Huguet et al., (2018)	✓	✓	✓	✓	✓	✓	✓	✓			✓	✗	✓	✓	SAT
Laberge et al., (2017)	✓	✓	✓	✓	✓	✓	n/a	n/a			✓	✗	✓	✓	SAT
Landsman et al., (2005)	✓	✓	✓	✓	✗	✗	✓	✓			✓	✗	✓	✓	SAT
Lofters et al., (2018)	✓	✓	✓	✓	✓	✓	✓	✓			✓	✗	✓	✓	SAT
Maciejewski et al., (2010)	✓	✓	✓	✓	✓	✓	✓	✓			✓	✗	✓	✓	SAT
McDonnell et al., (2022)	✓	✓	✗	✓	✓	✓	✓	✓			✓	✓	✓	✓	SAT
McMorrow & Zuckerman (2014)	✓	✓	✓	✓	✓	✓	✓	✓			✗	✗	✓	✓	SAT
Murayama et al., (2021)	✓	✓	✓	✓	✓	✓	n/a	n/a			✓	✗	✓	✓	SAT
Nishi et al., (2012)	✓	✓	✓	✓	✓	✓	n/a	n/a			✓	✗	✓	✓	SAT
Nolan & Layte (2017)	✓	✓	✓	✓	✓	✓	✓	✓			✓	✓	✓	✓	SAT
Peikes et al., (2018)	✓	✓	✓	✓	✓	✓	✓	✓			✓	✗	✓	✓	SAT
Phillips et al., (2014)	✓	✓	✓	✓	✓	✓	✓	✓			✓	✗	✓	✓	SAT
Ralston et al., (2009)	✓	✓	✓	✓	✓	✓	n/a	n/a			✓	✗	✓	✓	SAT
Supulveda et al., (2016)	✓	✓	✓	✓	✓	✓	✓	✓			✓	✗	✓	✓	SAT
Strumpf et al., (2017)	✓	✓	✓	✓	✓	✓	✓	✓			✓	✗	✓	✓	SAT
Wang et al., (2015)	✓	✓	✓	✓	✓	✓	✓	✓			✓	✗	✓	✓	SAT
Wherry & Miller (2016)	✓	✓	✓	✓	✗	✗	✓	✓			✗	✗	✓	✓	SAT
Yin et al., (2008)	✓	✓	✓	✓	✓	✓	n/a	n/a			✓	✓	✓	✓	SAT
Zhang et al., (2009)	✓	✓	✓	✓	✓	✓	✓	✓			✓	✗	✓	✓	SAT

*Results of the associated study can be found in the 'Study characteristics tables', see Tables 3 through 8.

Supplementary Table S3. Quality Appraisal of Qualitative Studies: CASP Checklist

Author (Year)	1	2	3	4	5	6	7	8	9*	10	Overall Appraisal
Burton et al., (2002)	✓	×	×	×	✓	?	✓	✓		✓	SAT
Ward et al., (2018)	✓	✓	×	×	✓	✓	✓	✓		✓	SAT

Supplementary Table S4. Quality Appraisal of Randomized Controlled Trials: CASP Checklist

Author (Year)	1	2	3	4	5	6	7	8	9	10	11	Overall Appraisal
DeVoe et al., (2015)	✓	✓	✓	×	✓	✓	✓	✓	×	×	✓	SAT
Horwitz et al., (2005)	✓	✓	✓	×	✓	✓	×	✓	×	×	×	CAUT
Persaud et al., (2021)	×	✓	✓	×	✓	✓	✓	✓	✓	✓	✓	SAT
Richards et al., (2014)	✓	✓	×	×	×	✓	✓	✓	✓	×	✓	SAT
Saluja et al., (2022)	✓	×	×	×	×	✓	✓	✓	×	×	✓	CAUT
